# Comparison of visual requirements and regulations for obtaining a driving license in different European countries and some open questions on their adequacy

**DOI:** 10.3389/fnhum.2022.927712

**Published:** 2022-09-30

**Authors:** Nina Kobal, Marko Hawlina

**Affiliations:** Eye Hospital, University Medical Centre Ljubljana, Ljubljana, Slovenia

**Keywords:** driving license, driving, medical examination, visual functions, vision impairment, visual acuity, visual field, contrast sensitivity

## Abstract

We reviewed the current state of knowledge regarding visual function and its suitability as part of medical examinations for driving licenses. We focused only on Group 1 drivers. According to previous studies, visual acuity, which is the most common test, is weakly associated with a higher risk of road accidents, with a greater role of visual field. The inclusion of the visual field test in medical examinations is therefore important, but the actual limit value is still unclear and further research in specific situations is needed. Color vision impairment was not found a threat to traffic safety. Contrast sensitivity decreases with age and is affected by abnormal eye conditions. Resulting glare can lead to an increased risk of traffic accidents during night driving in the elderly and others with conditions that impair contrast sensitivity. However, the universal cut-off limits have not been established either. The current European Union (EU) regulations therefore reflect minimum common denominator across the member states which may not entirely translate to optimal driving safety. Due to these open questions, standardized testing in simulators or on polygons that simulate real life conditions would be needed to better determine safe limits of visual function in different conditions. As there is a need to have better standardization across Europe regarding the requirements and rules regarding driving licenses in European countries, we first analyzed existing rules and compared them with each other, also in terms of deviations from the EU directive itself. We reviewed the literature in this field and prepared proposals for a more optimal regulation of the rules in the future. Particular attention is paid to the new method of examining the visual field that was created to respect the European directive. The paper can serve as a basis of information for research teams to design further protocols, as it gathers research findings to date on the importance and impact of various visual functions on driving safety, as well as a starting point for a debate on revising existing rules for obtaining and maintaining licenses, as it compares the current regulations in European countries and differences between them.

## Introduction

Driving motor vehicles is the primary mode of transportation in most countries. It enables an individual to fulfill daily obligations, so it is an important part of the life of most citizens and thus also affects the quality of it. Depression, social isolation and poor access to health care were more common among non-holders of driving licenses. It also causes greater dependence on the public or some other form of transport, which may be associated with higher costs and is also not available in all regions, especially in rural areas (Owsley and McGwin, 1999). Visually, we receive about 90% of all sensory information that is important for driving a motor vehicle (Honavar, 2019). Good eyesight is therefore undoubtedly the basis for safe driving in traffic. Eye diseases and irregularities most often affect perception disorders and can thus create difficulties with performing tasks, important for save driving, in individuals. Central vision may be impaired, or loss of peripheral visual field may occur. Perception is hampered by ocular surface diseases, intense glare, impaired eye movements, double vision, poor light adaptation, photophobia and pain, as well as sagging upper eyelids. A safe participant in traffic must be able to see far ahead, assess the shape, size, position, and mobility of various objects in their field of vision. Additional risk of a road accident is posed by external factors that are not influenced by the driver, such as poor visibility due to inadequate lighting, adverse weather conditions, unexpected traffic regulations, other road users etc. It is important to note that also good behavior, which is not tested, is of big importance in traffic. Good vision is important but irrelevant without behaving adequately. Younger and older drivers have a higher risk of accidents than middle-aged drivers. In young drivers, the cause is mainly alcohol and drug use, while in older people, health problems prevail, among which eye diseases are the leading cause (Owsley and McGwin, 1999).

## Visual functions important for the driver

### Visual acuity

Measuring visual acuity gives us information about central vision. To achieve optimal visual acuity, refractive errors need to be corrected. Visual acuity is a simple quantitative method of checking photopic visual function. It is usually checked with the help of tables (Snellen) with black marks on a white background for each eye separately, as well as with both at the same time. Ergoophthalmic devices are used in the daily practice of occupational medicine physicians in the examination of drivers’ vision, while testing conditions of these may differ from Snellen tables.

Visual acuity is thus a key function for distinguishing fine details. In traffic, it is crucial to identify objects on and along the road and to decipher traffic signs. Maximum visual acuity is achieved in daylight, when the sky is covered with clouds. The ability to see accurately decreases both in very bright light and in the dark. Acuity can also decrease with age due to opaque optical media, the most common causes of which are lens opacities (cataracts), vitreous disorders, ocular surface disease, and degenerative changes in the cornea. Age-related macular degeneration, glaucoma and diabetic retinopathy are among the most common causes of visual impairment at the posterior segment of the eye. Visual acuity deteriorates with age due to all these factors, which can be improved to some extent by increasing the brightness.

Visual acuity screening is probably the most common and fundamental method of examining vision as a part of medical examination to obtain a driver’s license in most countries around the world. Although visual acuity is a ubiquitous screening test when applying for a driving license, many other aspects of visual function and vision processing are undoubtedly involved in supporting effective vehicle control (Owsley and McGwin, 2010).

Despite the importance of visual acuity for distinguishing details, studies have not confirmed a strong association between moderately reduced visual acuity and reduced ability to drive safely. Reduced visual acuity is the most evident in poor recognition and understanding of traffic signs, avoidance of driving in poor conditions and ability to avoid road hazards. However, in the study performed by Higgins et al. (1998) many other components of the overall driving were not affected by the decreased acuity. Participants were able to navigate the vehicle through test courses under all conditions, although more slowly when having low acuity. Performance on the gap clearance task was not correlated to visual acuity level and performing on the maneuvering task was only partially correlated. The series of studies done by Hills and Burg (1977) showed no correlation between visual acuity and the risk of having an accident in the group of young and middle-aged drivers, however, a weak association was found amongst older drivers. This finding was supported by some other studies (Zhang et al., 2007; Owsley et al., 2015; Salvia et al., 2016). In a recent Alvarez-Peregrina et al. (2021) study, significant differences were found regarding the risk of having an accident according to visual acuity (*p* < 0.001), with the participant’s median age of 46. However, two earlier studies which had a big sample size and were done by Rubin et al. (2007) and Cross et al. (2009), did not find any correlation between visual acuity and the risk of accident.

From this we can conclude that it is somehow paradoxical that visual acuity is the most frequently investigated visual function, but at the same time it has no strong connection in identifying high-risk drivers (Owsley and McGwin, 2010). There are also some explanations for this finding. Acuity tests using standard tables may not be a good indicator of an overall assessment of an individual’s ability to drive (Ginsburg, 1987; Freeman et al., 2005). Driving on the road and through intersections involves the simultaneous use of central and peripheral vision and requires monitoring of primary and secondary tasks, all during a rapidly changing environment, where critical events occur with little or no warning. Visual sensory tests do not usually involve these stimuli but seek to minimize distractions and secondary tasks. Acuity tests the ability of the eye to focus on image, therefore it is a measure of quantity, not quality of the vision (Ginsburg, 1987). Furthermore, the details which are relevant in traffic are larger than print size letters. Drivers with reduced acuity and still recognize most of the objects on the road which are important for safe driving. Thus, it is not surprising that conventional vision test such as visual acuity is not a strong predictor of collision risk, as mentioned in numerous studies (Owsley and McGwin, 1999, 2010). In addition, the efficiency of vision is affected not only by the sensory and physiological parts of the optical apparatus, but also by the central processing of information. This is also the basis of the second aspect, that the relationship between visual acuity and driving performance cannot be properly considered without taking into account other aspects of visual performance at the same time (Owsley and McGwin, 2010). Therefore, the current cut-off of visual acuity will need to be reassessed under real conditions.

### Visual field

The visual field is defined as the area within which information can be detected with the immobilized head and eyes directed at the fixation point (van Rijn, 2005). With the distance of the stimulus from the macula, visual acuity decreases rapidly, which corresponds to the arrangement of the photoreceptors. The cones in the center allow us sharp central vision, and the peripherally arranged rods serve to detect objects and movements around us. Peripheral visual acuity is only 3–5% of the central, which is good enough to detect the object and direct the gaze toward it.

When we drive, we need to see well the object that is at the center of our view, but at the same time we need to clearly perceive the objects that are around the center and on the sides. Good peripheral vision thus allows us to see cars in adjacent lanes or pedestrians on the sidewalk. Overlapping the visual field from the left and right eye, the so-called binocular visual field, is also important for driving. The most important information while driving is within the central 20° of visual field (van Rijn, 2005). Binocular scotomas in central vision are thus regarded as a contraindication for driving motor vehicles. In addition to the central visual field, the left and right horizontal area, is also extremely important, for example for changing lanes. The vertical visual field is often limited by vehicle parts and is less important for driving safety.

The visual field is measured by perimeter. This can be static or kinetic. In static perimetry, the measurement is performed in automated fashion. The light targets of different intensities appear in random places in the visual field of a subject. In kinetic perimetry (Goldmann), however, the location of light stimuli of different intensities is perceived while moving, usually from the periphery toward the center. Kinetic perimetry is more dependent on the testing conditions and communication between investigator and patient and can sometimes miss minor central or paracentral scotomas. While the peripheral visual field is usually well charted, the accuracy for detecting central scotomas is lower than in static perimetry. Portare Working Group (2009) suggested using confrontational method at the beginning but refer a driver to Estermann binocular visual field test in case of any doubt. The Eyesight Working Group, however, stated that verification by the confrontational method has low sensitivity and specificity, so it is not suitable for screening (van Rijn, 2005). Drivers’ examinations usually use electronic computer-guided perimeters for screening of the visual field, usually with light signal that appears 35°, 55°, 70°, and 85° temporally on each eye. However, in case of deviations, Goldmann or static perimetry is needed (Gruber et al., 2013). The Eyesight Working Group (van Rijn, 2005) recommended checking 100 points in the horizon, of which at least 25 should be in the central 20° of the field, and the intensity of light signals should increase toward the periphery. Luminosity is expected to be higher in the elderly. Individual scotomas (which may be due to a well-tolerated disease) should be considered individually in the assessment of ability, although a longer procedure is required. Scotomas in the peripheral visual field can also be the result of wearing glasses during the examination and their frames. Also, false positive results occur when performing measurement without suitable correction. The first measurement usually has poorer results due to learning effect, so they advise to repeat the measurement in the case of doubt.

The boundaries of the normal visual field change depending on the position of the eyeball in orbit or on the shape of the orbit and nasal root. Changes also occur with age. The volume of fat in the orbit is reduced, the eyeball lies deeper, which can result in slightly narrowed outer boundaries of the visual field. In old age, the upper eyelid often sags slightly, resulting in a narrowing of the visual field in the upper part. Pupils are also narrower in old age than in youth due to parasympathetic predominance, and pupil width may also affect the width of the visual field. However, diseases such as glaucoma, age-related macular degeneration, diabetic retinopathy, retinal and optic vascular disorders affect the visual field more than physiological changes that are natural and expected in old age (Gruber et al., 2013). It is important to point out that eye diseases are not the only ones that affect the visual field. Stroke is the most common cause (in 52–70% of cases) of homonymous hemianopia in adults, followed by traumatic brain injuries (14%) and tumors (11%) (Zhang et al., 2006; Goodwin, 2014). Those patients may also have other consequences (e.g., memory problems, attention impairments, problems with balance and movements, etc.) that can all affect driving and must be considered. Portare Working Group (2009) suggests doing clinical test that is similar to confrontational method when testing visual field.

Johnson and Keltner (1983) found that drivers with a field loss in one eye were not significantly different from full-field binocular drivers, whilst drivers suffering from a severe binocular vision field loss had double the rate of accidents compared to drivers with normal visual field. Wood and Troutbeck (1994) conducted an experiment in which they used modified swimming goggles to restrict visual field size while participants needed to complete various driving tasks on a closed-road circuit. Reducing the field size resulted in significant reduction in detecting on-road obstacles, a reduced ability to detect peripheral objects (pedestrians and road signs), a decrease in the ability to maintain a steady position within the lane and a speed decrease. Similar was observed by Owens and Tyrrell (1999) who found steering to be sensitive to a reduced field size but not to blur or luminance. It was also proven that loss of peripheral visual field caused longer search times, more fixations with shorter duration, more errors and difficulties with driving maneuvers in comparison with drivers with normal visual field (Coeckelbergh et al., 2004; Parker et al., 2011; Bronstad et al., 2015; Kübler et al., 2015). In an on-road driving evaluation done by Elgin et al. (2010), 41% of drivers with homonymous hemianopia had trouble controlling the vehicle position, 36% had problems adjusting their speed to traffic conditions, 27% did not respond adequately to unexpected events, and 27% had unusually bad driving maneuvers. Similar problems were found among participants with homonymous hemianopia in the study by de Haan et al. (2014). Despite these problems, drivers were considered safe to drive by driving rehabilitation specialists in 87–88%, respectively (Wood et al., 2009; Parker et al., 2011; Alvarez-Peregrina et al., 2021). In the study by de Haan et al. (2014) 54% of participants with were evaluated as fit to drive. Furthermore, several studies found limited (Wood et al., 2009; Bowers, 2016) or no (Lachenmayr, 2006; Alvarez-Peregrina et al., 2021) correlation between people with homonymous defects and higher rate of accidents.

Different results from studies could be the consequence of different definitions of impairment in the visual field because the standards for that vary in different countries. Other factors could be the differences in participant’s characteristics, size of the sample, inclusion criteria, assessment procedures, visual function deficits, as well as traffic conditions and regulations (de Haan et al., 2014; Alvarez-Peregrina et al., 2021).

As visual field tests measure target sensitivity on a simple background without head and eye movements, this is in direct contrast to the complexity of driving, where head and eye movements are used to inspect the environment.

Research has shown that some patients with visual field loss can partially compensate for such limitations by increasing head and eye movements and fixations in the field of visual field loss, which makes their driving performance similar to that of normal subjects, while others display obvious vehicle control problems (Tant et al., 2002; Coeckelbergh et al., 2004; Wood et al., 2009; Parker et al., 2011; Papageorgiou et al., 2012b). Initial compensatory strategies include dislocated gaze position and a distorted fixation ratio toward the blind side (Papageorgiou et al., 2012b; Biebl et al., 2022). Compensatory behavior becomes even more evident during the more demanding tasks (Papageorgiou et al., 2012b). Bowers et al. (2014) observed that drivers with homonymous hemianopia demonstrated compensatory head scan patterns but not scan magnitudes which resulted in detection failures on the blind-side. Results from the study by Alberti et al. (2017) suggested that only a minority of patients with homonymous hemianopia are able to spontaneously adapt their blind-side scanning. Scanning training is therefore beneficial for those individuals as it helps them use their residual vision more efficiently and should be part of the treatment (Bowers et al., 2014; Goodwin, 2014). This emphasizes the need for driving lessons (on-road or in a simulator) to be included in the training program preceding the Practical Fitness to Drive Test (de Haan et al., 2014). Training should include activities that improve general vision attention skills, increase the number and amplitude of saccades into the blind-side and develop a more organized pattern of eye movements (Goodwin, 2014). Treatment can also include or be upgraded with the prismatic correction to expand the remaining visual field (Goodwin, 2014). A clinical trial demonstrated that treatment with prisms improved mobility and avoidance of obstacles (Bowers et al., 2014). Driving with prisms enables many drivers with visual field deficits to obtain their driving licenses. Furthermore, a driving license should not be rejected solely on having the visual field loss but on the inability to compensate for it.

A recent study, done by Andersson et al. (2022), found that self-assessed skills did not predict driving performance well. Groups with different stages of visual field loss rated their skills similarly, therefore self-rating of driving abilities cannot be reliably used.

Papageorgiou et al. (2012a) stated that no vision-related parameters are suitable to predict accident involvement but an individualized approach that takes into account compensatory strategies (eye and head movements) as well. Regarding self-reporting, older drivers with poorer results on the tests of contrast sensitivity and visual field are more likely to stop driving on their own decision (Freeman et al., 2005; Thorslund and Strand, 2016).

Visual requirements for driving in European countries generally follow the standards of the European Union (EU) Directive 2006/126/EC, and according to the requirements for holders of Group 1 driving licenses (car and motorcycle), the binocular visual field should be at least 120° horizontally, the extension should be at least 50° left and right and 20° up and down, and no defects should be present within a radius of the central 20°. Still, important ambiguities can be found in implementation of the European standards. Furthermore, there was no mutual agreement or recommendation for the appropriate perimetry algorithm for assessing the visual field requirements and there is no uniform approach regarding interpretation of the results. It is, for example, not clear how many missed dots or degrees defines a defect within the central 20°. Therefore, the »gray zone« around the current EU standards may be relatively large (Gruber et al., 2013). These ambiguities lead to differing practices in enforcing visual field regulations, which ultimately challenges the fundamental right of European drivers for legal equality. To put focus on this topic, a comparative international survey on 15 patients’ visual field defects was done. The research question was whether these patients fulfill the visual field requirements in different European countries. Each of the 15 cases were presented with diagnosis, threshold perimetry and binocular Esterman results. The results of the survey (Sudmann et al., 2022), showed that there were only a moderate level of agreement for determination of pass/fail criteria in different countries using suprathreshold and Esterman testing.

As Esterman test was never developed especially to assess visual functions for driving, it was still most often used in this area because it is a binocular test and was close to covering EC Directive requirements but not all of them. A novel binocular algorithm that complies with more of the requirements of EC Directive was recently developed and been made available for Octopus 900 (Jørstad et al., 2021). Similar to the Esterman’s algorithm, the new traffic perimetry algorithm is a binocular supra-threshold test with approximately 120 test points, but there are several factors that distinguish the two. First, to unequivocally define a positive result within 20°, the new perimetry algorithm has 37 equidistant test points within 20°, including seven within 7° (the Esterman program has 24 test points within 20°, of which eight are located superiorly, 16 inferiorly, but none within 7°). However, it is important to mention that the number of test points within specified areas differ between Esterman programs in different perimeters (Bro, 2022).

Second, the new perimetry algorithm presents a supra-threshold stimulus that dynamically follows the physiological hill of vision. By way of comparison, the Esterman’s algorithm presents a 10 dB stimulus throughout the visual field. This value is not an absolute measure but vary with the maximum light stimulus of the perimeter in question (Bro, 2022). As the sensitivity to light under photopic conditions is highest centrally and gradually decreases toward the periphery, the fixed Esterman’s stimulus introduces a bias regarding relatively lower sensitivity for central than peripheral visual field defects. The problem of this test is that it still does not take into account compensatory eye and head movements.

If this new test (Jørstad et al., 2021) will become uniformly applied in all countries, we may obtain harmonized test strategy, whilst further research would still be needed to evaluate the differences between the formal cut-off criteria and of the effect of particular loss of visual field on a simulator or in real road conditions. As mentioned above different boundaries for a minimal required visual field size are applied in different countries. None of these values are based on scientific evidence or clear rationales so far (de Haan et al., 2014). The official on-road driving test is by definition a valid test to evaluate whether persons with impairment are fit to drive, since the evaluation takes place in the same situation that is being evaluated (driving on the road) (de Haan et al., 2014). Therefore, more practice with actual driving tests (on the road or in the simulator) to discriminate between safe and unsafe drivers with different types of visual field loss would need to be implemented in EU-countries with the use of Practical Fitness to Drive Tests.

### Depth perception

Depth perception (stereoscopic vision) is the ability of the eye to see objects in three dimensions and estimate the distance to the object. This feature is important in situations when we are driving behind a vehicle and for assessing how fast a vehicle is approaching us. One of the conditions that may contribute to the loss of stereoscopic vision is amblyopia, a frequent condition, characterized by a reduced best corrected visual acuity in the absence of a structural cause. The condition may lead to the suppression of the amblyopic eye and consequently to the loss of stereoscopic vision (Webber et al., 2020). There are many causes for amblyopia, including strabismus, neglected refractive errors, diseases that obstruct the visual axis (e.g., congenital cataracts) (Grant and Moseley, 2011; Webber, 2018).

Although regulations generally allow stereoscopically deficient individuals to drive, previous studies have revealed discordant findings regarding their on-road performance and safety (Owsley and McGwin, 2010; Levi et al., 2015). Many interviews and questionnaires have studied the impact of amblyopia and similar conditions on patients’ ability to drive (West et al., 2003; Kumaran et al., 2019). So far, there is no scientific evidence that having amblyopia is a contra-indication for driving.

Ergoophthalmological apparatus for examining visual functions is commonly used for the measurement of depth perception in driver examinations. However, the limit value for the Group 1 drivers has not yet been defined and there was no particular test tested in real-life conditions (van Rijn, 2005).

### Color vision

Color vision problems are not usually the reason why an individual would not be able obtain a medical certificate for a driver’s license, especially if it is not related to another eye pathology. Poorer or absent recognition of red and green was not shown to be a major problem, as these colors are mostly used in traffic in established patterns (e.g., traffic lights). A concern may be very poor or not recognizing red, as such individuals may have slower reactions to red brake lights, which are more unpredictable in traffic. It is also important to know that such individuals are mostly not color-blind but only have a reduced ability to recognize colors (Owsley and McGwin, 2010).

For driving licenses examinations of the first group, the rules do not specify the methods of measurement and the required minimum criteria (van Rijn, 2005).

### Contrast sensitivity

Contrast sensitivity allows us to distinguish objects and shapes that do not differ much in brightness or color from the background and that have a blurred or indistinct edge.

According to Pelli-Robson test, the prevalence of contrast sensitivity of < 1.25% was very rare in young people, between the age of 65 and 74 it was present in 1.7% of cases, and in 6.3% in the group of older than 75 years. The prevalence of contrast abnormalities was thus higher than the prevalence of visual acuity and field abnormalities (van Rijn, 2005).

Research showed that the contrast sensitivity was the most perceived factor amongst drivers, which led to difficulties in driving during the day and night (Rubin et al., 1994). Contrast sensitivity can detect cataract and poor contact lenses that go undetected by visual acuity testing (Ginsburg, 1987). Unlike visual acuity, contrast sensitivity does relate to visual performance in real-life situations (Ginsburg, 1987). Thorslund and Strand (2016) stated that even self-reported contrast sensitivity was a better predictor than visual acuity alone. Owsley and McGwin (2010) found that contrast sensitivity in the elderly with the presence of mature cataracts has been strongly associated with recent collision history. Additionally, in individuals with developed cataracts, surgery reduced the likelihood of a collision by 50% compared to those who did not undergo surgery. The greatest impact on this was mainly the improvement in contrast sensitivity after surgery (Owsley et al., 2002; Wood and Carberry, 2006; Owsley and McGwin, 2010). However, some other studies did not find important correlations between contrast sensitivity and safe driving (Cross et al., 2009; Alvarez-Peregrina et al., 2021). This could be due to different ages of participants in the studies as having older participants can mean higher occurrence of cataract among them.

According to the illuminance, we use three common types of light conditions. There are three common types of light conditions; photopic (>10.0 cd/m^2^), mesopic (<10.0 and > 0.01), and scotopic (<0.01). Due to road and traffic clearing, we drive most of the time in mesopic and not scotopic conditions during the night. Wood et al. (2009) and Owsley et al. (2015) state that the contrast sensitivity test under photopic conditions is a better predictive factor for the success of recognizing traffic signs and pedestrians at night than the visual acuity measured under photopic conditions. Drivers with reduced contrast sensitivity completed fewer kilometers per year and fewer long-distance journeys and trips per week compared to those with normal contrast sensitivity, even after adjusting for other factors (Sandlin et al., 2014). Reducing acuity alone (at the level 20/40 and 20/80) did not significantly reduce reaction time or the deceleration needed to stop before the collision point. Adding a contrast sensitivity loss to an acuity deficit increased reaction time and the deceleration required (Wood and Troutbeck, 1994; Higgins and Wood, 2005; Wood et al., 2009). Contrast sensitivity should be considered when talking about someone’s fitness to drive, especially in early stages of some ocular diseases (e.g., cataract) when contrast sensitivity may be already impaired while high contrast acuity is still unimpaired (Swan et al., 2019). Similar was found in study by Jones et al. (2022) who observed that contrast sensitivity and low contrast visual acuity predict ability for night driving in a manner that conventional high contrast acuity does not.

The contrast sensitivity investigation therefore adds an important information about the ability to drive. The problem is the fact that it is not yet known which limit value would still be acceptable for safe driving, nor are the limit values defined in the regulations. Jones et al. (2022) stated that contrast sensitivity measurements can be made on a driving simulator and the results are in good agreement with conventional methods (e.g., Optovist). An additional problem is that the rules do not specify the requirements and norms for performing the measurement itself. Thus, the result of measurements on one apparatus may not be comparable to measurements made with other devices in other centers (van Rijn, 2005). Therefore, a comparable method of contrast sensitivity testing should be established and standardized.

### Sensitivity to glare

Glare occurs when the retina is illuminated by a strong light source (e.g., a headlight) at dusk or at night. The eyes then need some time to get used to the subject again, with older people having this time significantly longer than younger drivers. Sensitivity to glare is one of the most frequently mentioned inconveniences of night driving in older drivers. There are several reasons why the elderly drivers are more sensitive to glare. Age-related changes in the optic media cause increased light scattering. This, in turn, increases the sensitivity to glare even in people with otherwise perfectly healthy eyes. On the other hand, the time required to adjust vision after a short but intense glare also increases with age. Factors affecting this time include lens optical density, sensory photopigment regeneration, and spherical aberrations (Owsley and McGwin, 1999).

While driving, glare occurs mainly due to incoming vehicles and street lighting. Lachenmayr (2006) pointed out that several studies have shown a link between glare sensitivity and night driving performance, where glare increases the risk of accidents, especially night collisions with other road users. Increased sensitivity to glare is also associated with slower object detection and slower speeds on dark and winding roads. Both are more pronounced in older drivers (Gruber et al., 2013). Seventy-five percent of people over the age of 70 were unable to distinguish contrast in glare conditions, which, however, does not mean they are unable to drive (Gruber et al., 2013). Puell et al. (2004) found that mesopic vision without glare begins to decline around age 51–60, while mesopic vision with glare begins to decline as early as in the age of 41–50. Therefore, glare sensitivity is an investigation that is particularly important for the assessment of severe disturbances that would have a significant impact on driving safety. However, due to different methodology, the uniformly established cut-off limit has also not been established.

In addition, more and more patients are recently being fitted with multifocal intraocular lenses, as this is becoming an internationally accepted standard for people who want to be independent of glasses after the age of 45 for both far and near. Because these lenses refract light differently, the glare test can scatter light more strongly, although these lenses allow for sufficiently clear vision at different distances and testing with light scattering devices did not show significant deviations from monofocal intraocular lenses (Cerviño et al., 2008; de Vries et al., 2008; Łabuz et al., 2016). Therefore, in these patients, glare and contrast sensitivity testing on occupational medicine devices is not appropriate for assessing the quality of vision. These patients, especially in the first months after surgery, may have slightly lower contrast sensitivity and dysphotopsias, but this is not a reason to limit night driving anywhere in the world. In addition, some types of LED headlights produce more glare effects than the others and research would be needed to standardize types of headlights that offer maximum safety with least glare effects.

### Night vision

Night driving requires more mesopic than scotopic vision, as there is always some light available when driving at night. With age, mesopic vision deteriorates and sensitivity to glare increases even in the absence of eye disease. Due to the growing number of older drivers, night vision problems are affecting a growing proportion of drivers.

Numerous studies have found a link between impaired mesopic vision or increased sensitivity to glare and impaired night driving, while links to some other tests (e.g., visual acuity and visual field) have not been found (von Hebenstreit, 1984; Lachenmayr et al., 1998; McGwin et al., 2000; Theeuwes et al., 2002). The relationship between photopic visual acuity, the most used test in assessing older drivers, and night driving ability has not been fully elucidated, but some authors state that visual acuity measured in photopic conditions is not in itself a good predictor of night driving ability (van Rijn et al., 2002; Wood and Owens, 2005). After the age of 65, mesopic visual acuity is expected to begin to decrease significantly. The drop is expected to be even greater in older drivers when lens opacities are already present (Gruber et al., 2013).

Visual acuity in mesopic conditions and sensitivity to glare thus prove to be important for determining night driving ability, but the limit value for driving ability is again not set and varies according to the type of testing. Important dilemmas regarding the introduction of different functional tests are set out in more detail in The Eyesight Working Group report (van Rijn, 2005).

### Double vision

Double vision can be caused by several ocular conditions, including astigmatism or corneal abnormalities, but it is most frequently caused by neurological diseases or injury. Double vision can lead to the detection of false images that make it difficult to drive safely, especially at night and on narrow streets. Portare Working Group (2009) suggests doing a simple »fix and follow« test of eye movement which involves holding a target object at arm’s length in front of the candidate. Double vision in the primary position can be a circumstance that prevents safe driving and should be a contraindication to issuing a driving license if it is not possible to eliminate double images with prisms (Righi et al., 2014).

### Monocularity

The perceptual abilities of a driver using only one eye are reduced accordingly. On the side of the practically blind eye, the visual field may be narrowed. If drivers who have only one healthy eye want to look to the affected side, they must turn their head. They also might have problems with reversing. Nevertheless, such an individual can participate in traffic after adaptation period. Optional adjustments, such as properly fitted and adjusted rear- view mirrors, which must be on the front on both sides (Gruber et al., 2013), could be useful in some of these cases. Real-life experiments did not demonstrate poorer driving performance in monocular drivers, while some simulation studies showed that drivers with sudden monocular vision were more likely to crash and drive off the road (McKnight et al., 1991; Wood and Troutbeck, 1994; Tijtgat et al., 2008; Adrian et al., 2019; Derhy et al., 2020; Molina et al., 2021). Restrictions are mostly placed on drivers who have recently become monocular because they may require some adaptation time (Dakroub et al., 2022).

## Comparison of regulations for obtaining a driving license regarding visual functions among different European countries

Due to the need for comparable security standards, the European Commission Directive 2009/113/EC defined the European visual standards for driving (that were previously described in Directive 2006/126/EC), which all EU member states must incorporate into their national laws. These rules were aimed at defining minimum criteria that EU member states should follow, whilst the criteria for individual member states may be stricter if they decide so. For comparison among selected European countries, we prepared comparative tables (Tables 1–4) regarding individual visual function, which present the currently valid criteria when examining vision for a Group 1 driving license and evaluate them according to the degree of deviation of their requirements from the requirements of the European Directive. Data on criteria used by these European countries were collected through a detailed review of the legislations currently in force, according to their available internet sources. We included all European countries for which it was possible to find official information about the regulations on the websites or from the available published sources in English. All the sources are collected in the Supplementary Table 1. We included the UK and Switzerland and Norway as well, although they are not member states of EU, however. they are in European states and should not deviate much from EU countries.

**TABLE 1:** European Union *visual acuity* requirements and comparison of the requirements among selected European countries according to the degree of deviation.

	EU directive (2006/126/EC)	At least 0.5 binocular (with or without correction)
**Countries with a small deviation or the same conditions as the EU directive**	**Hungary**	At least 0.5 binocular, if the person is monocular, it must be at least 0.5 (with or without correction)
	Austria	At least 0.5 binocular (with or without correction)
	Slovenia	At least 0.5 binocular (with or without correction)
	France	At least 0.5 binocular, if the person does not see on one eye or the acuity on this one is less than 0.1, it must be at least 0.5 on the other (with or without correction)
	Croatia	At least 0.6 binocular, at least 0.2 on the worse eye, the difference between them should not be bigger than 3D (with or without correction)
	Switzerland	At least 0.5 on the better eye, at least 0.2 on the worse eye, if the person is monocular, the acuity must be at least 0.6 (with or without correction)
	Denmark	At least 0.5 binocular (with or without correction)
Countries with a marked deviation from the EU directive -in the stricter direction:	Italy	At least 0.7 binocular, at least 0.2 on the worse eye, the difference among them should not be bigger than 3D (with or without correction)
	Germany	First, the visual acuity is checked, which must be at least 0.7 binocular, if this is not possible, a more extensive ophthalmological examination is done, where the visual acuity must be at least 0.5 binocular (with or without correction)
Countries with marked deviation from the EU directive -in less strict direction:	The Netherlands	Candidates complete the Health Declaration questionnaire before taking the exam and upon extension. If they indicate that they have certain vision problems, they must undergo an ophthalmological examination, where the acuity must be at least 0.5 binocular (with or without correction). The driving test starts with reading a license plate of the car at the 25 m distance (requiring visual acuity around 0.7). If one fails this test, one needs to undergo ophthalmological examination.
	United Kingdom	At the beginning of the practical part of the exam, one must read the license plate from 20 m. If one fails, obligatory ophthalmological examination follows, where the visual acuity must be at least 0.5 binocular (with or without correction)
	Iceland	Opportunity to self-certify that no visual impairment is present. If not, visual functions are not tested. If there are problems, same criteria as in EU directive are used.
	Norway	Opportunity to self-certify that no visual impairment is present. If not, visual functions are tested at the driver’s test by reading a road sign at 20 m distance.
	Sweden	If nystagmus is present, visual acuity has to be of 0.5 when the head is turned 30°.

EU, European Union; D, diopter.

**TABLE 2:** European Union directive *visual field* requirements and comparison of the requirements among selected European countries according to the degree of deviation.

	EU directive (2006/123/EC)	Horizon at a visual field of at least 120°, the extension must be at least 50° to the left and right and 20° up and down. There must be no scotomas in the radius of the central 20°
Countries with a small	Slovenia	Same requirements
derogation or the same	Italy	Same requirements
conditions as the EU directive	Hungary	Same requirements
	Austria	Same requirements
	Switzerland	Same requirements
	France	Same requirements
	Denmark	Same requirements
Countries with a major	Croatia	Mentions only the criteria that there should be no scotomas in the radius of the central 20°
deviation -in less strict direction:	The Netherlands	The field of vision is examined only if there are deviations in the questionnaire, otherwise not (in case the person needs an extended ophthalmology examination, the same criteria as in the EU directive are observed)
	Germany	Visual field is tested only if the candidate does not have adequate acuity on the initial vision test (then the same criteria as in the EU directive are observed)
	United Kingdom	Visual field is tested only if the candidates have to undergo an extended examination because they failed to read the license plate (then the same criteria as the in the EU directive are observed)
	Iceland	Opportunity to self-certify that no visual impairment is present. If not, visual functions are not tested. If there are problems, the same criteria as in the EU directive.
Countries with a major	Norway	If there is suspection of visual field deficit, Esterman perimetry is performed. No more than four missed test points within 20° are allowed.
deviation -in stricter direction:	Sweden	In threshold perimetry, all corresponding test points within 20° need to be at least 10 dB and all corresponding test points within 10° need to be 20 dB or more. Not more than two adjacent missed points within 120° horizontally and 40° vertically in Esterman perimetry.

EU, European Union.

**TABLE 3:** Requirements of the European Directive regarding *additional investigations* of visual functions and comparison of the requirements of selected European countries according to the degree of deviation.

	EU Directive (2006/123/EC)	Attention should be paid to vision in low light, sensitivity to glare and sensitivity to contrasts, diplopia and other visual functions that may compromise safe driving. Methods and criteria are not specified.
According to additional investigations strict countries:	Italy	In addition to acuity and visual field, they also examine contrast sensitivity, night vision is examined.
	Denmark	Night vision, contrast sensitivity, glare sensitivity are suggested to be part of examination but are not requested by the law for group 1.
According to additional investigations less strict countries:	Croatia	Color vision
	Hungary	Color vision
	Slovenia	In the basic examination: only acuity and visual field, if not appropriate in the extended examination all the pertinent tests are done.
	Austria	In the basic examination: only acuity and visual field, if there are deviations, double vision, contrast sensitivity, and glare sensitivity are checked.
	Germany	Initially only acuity, if not appropriate in the extended examination all the pertinent tests are done.
	The Netherlands	They fill in a questionnaire, if they indicate that they have problems, all the pertinent tests are done (they have to indicate if they are visiting any medical specialist; if so, each of the specialist have to provide information about your
	United Kingdom	Initially, just reading the license plate. If there are problems with it, an ophthalmological examination where all the pertinent tests are done.
	France	Initially, only acuity and visual field, if the criteria are not meet, then night vision, double vision, contrast sensitivity, and sensitivity to glare are checked.
	Iceland	Opportunity to self-certify that no visual impairment is present. If not, visual functions are not tested.
	Norway	Only visual acuity and visual field.
	Sweden	Only visual acuity and visual field.

EU, European Union.

**TABLE 4:** Requirements of the European Directive on the *renewal of driving licenses* and vision check-ups and a comparison of the requirements of selected European countries according to the degree of deviation.

	EU Directive (2006/123/EC)	From 19/01/2013: The driving license is valid for 10 years. Individual member countries may decide to extend it to 15 years. Member countries may require a medical examination (usually only if any medical restriction is identified at the first examination before getting a license). The Directive allows the criteria to be stricter in each member country.
Countries with requirements similar or less strict than the EU directive	Slovenia	Drivers, who do not have a limited validity of driving license due to health reasons, must attach a medical certificate when renewing a license after the age of 70 (or 80 if they have received a license by the old law).
	Austria	Renewal every 15 years, no medical examination required. If there are ophthalmic problems at the first check-up when license is being issued, the person gets permission for a shorter period and needs to medical examination at every renewal.
	Germany	Driving licenses are extended to 15 years, no medical examination is required. If there is a time limit in the medical certificate, then a medical examination must be performed at specific periods.
	Switzerland	At the age of 18, they get license for 3 years, if during this time everything is fine, there is no need to renew it up to the age of 75, then the medical examination and renewal is needed every 2 years.
	France	They need to extend the driving license every 15 years, if they have no problems, medical examination is not necessary. There was no age limit written for medical re- examining if the person does not have problems. If you have an accident, you must take a vision test.
	The Netherlands	They extend it every 10 years, except after the age of 70 every 5 years, they have to undergo a medical examination every 5 years after the age of 75.
	Denmark	They extend their driving license every 15 years. Over age of 70–71: every 4 years, 72: 3 years, 72–79: every 2 years. Over age of 80 every 1 year. After the age of 75, they need a medical certificate for renewal.
	United Kingdom	They extend the license every 10 years, after the age of 70 every 3 years. Medical examinations are not obligatory if there are no problems. They sign a declaration that they are fit to drive, and are otherwise responsible for themselves to make an examination, if they are not. If one has an accident and it is found out that one does not have good eyesight, penalties are high.
	Iceland	After 65 years of age, a physician has to certify vision at renewal, after 72 years of age every second year. After 80 years of age every year.
	Norway	After 75 years of age, a physician has to certify vision at renewal, after 78 years of age every second year.
	Sweden	No requirements
Countries with stricter requirements	Hungary	The driving license is extended to 10 years until the age of 50, between the age of 50 and 60 for 5 years, between 60 and 70 for 3 years and after the age of 70 for 2 years. A medical examination is always required.
	Italy	Every 10 years, after the age of 50 every 5 years, after the age of 70 every 3 years, after the age of 80 every 2 years (a medical certificate is required for each extension).

EU, European Union.

As it can be seen in Table 1, Croatia, Italy and Switzerland have additionally defined criteria for the worse eye. Most countries do not have a defined method in the legislation to check visual acuity, except for United Kingdom, which mention the use of Snellen tables. Italy and Germany deviate the most in the stricter direction. Italy requires at least 0.7 binocular and 0.2 on the worse eye, while Germany has an entry criterion of 0.7 binocular, but if failed, ophthalmology exam must be undertaken after which, binocular visual acuity with or without correction must be at least 0.5, which is equal to the EU criterion. The Netherlands, Iceland and Norway are receding in the other direction, relying on the responsibility and sincerity of the candidates with a questionnaire on health problems. Only those candidates who indicated in the questionnaire that they have vision problems or have already been treated by an ophthalmologist are referred for an examination of visual functions. However, in the Netherlands they do quick check of acuity with reading a license plate before staring the driving test. Similar situation is present in United Kingdom, where they rely on license plate reading as basic testing, which probably overlooks some with poorer visual acuity.

From the Table 2, we can summarize, that countries that have legalized a visual field examination as a part of a basic screening of each candidate adhere to the same criteria as the EU directive. Deviations are mainly present in countries where the visual field is not part of the basic examination, but only part of the extended ophthalmological examination, if deviations occur in the basic one (which usually consists of only visual acuity test). In the legislation, the method for the visual field test is defined by Hungary and Denmark (the confrontational method is sufficient for the basic inspection, a closer examination is needed if they fail the basic inspection) and Germany, which states that in the case of visual field defects it is necessary to perform an investigation with Goldmann III/4 target. In everyday practice, in most countries, the device on which several visual functions can be tested is used initially (e.g., Optovist, Rodatest, or similar device). In case of deviations, perimetry with Goldmann perimeter should be performed. Sweden and Norway are stricter in this matter as they specify the number, position and threshold value of test points in their legislation (Bro and Lindblom, 2018). They give more importance to visual field deficit compared to acuity which is consistent with the literature about visual field deficit affecting driving performance more than high contrast visual acuity. In Norway, they use Esterman perimetry. In Sweden, physicians are allowed to test the field of vision with Donders test. All others must use an instrumental visual field test. Most commonly horizontal field of 120–180 degrees is tested whilst vertical field is not tested. If no visual impairment is suspected, a simple instrument testing the visual field borders is used. In the event of visual problems, threshold perimetry, for example, Humphrey 24–2 must be performed. In the peripheral field, testing is carried out using the binocular Esterman programme in an automated perimeter (Bro and Lindblom, 2018).

To summarize the Table 3 of the countries considered, only Italy tests all visual functions already at the initial examination. Denmark suggests performing all the tests at every examination, but only visual acuity and visual field are requested by the law for Group 1. Until recently, in Slovenia, all candidates were checked for depth perception, color vision, contrast sensitivity, phoria and glare sensitivity already in the basic examination. However, that was changed in February 2022, when the new regulations entered into force that demand only visual acuity and visual field testing. All other countries have mostly only visual acuity and perhaps a visual field in the basic examination, whilst all other investigations are part of the extended examination in case of deviations at the basic one. In their legislations, most countries do not have defined standard methods that should be used in investigations. In practice, most of them use various devices (e.g., Rodatest, Optovist, or similar), on which all the mentioned functions can be examined. Only Hungary and Croatia have mentioned in the legislation that Ishihara tables should be used for the color vision test.

Based on Table 4, Slovenia, Switzerland, Iceland, Norway, and the Netherlands are the countries that have an age-limit at which, despite having no vision problems in the past, regular vision tests are needed. In most countries, it is not necessary to perform a visual function check-up if there were no problems at the examination when the driving license was issued. However, vision tests must be performed at each extension in Italy and Hungary. Sweden is the only country without periodic testing for elderly (Bro and Lindblom, 2018). Consequently, a 75-year old patient with age-related dry macular degeneration and bilateral acuity of 0.4 may continue to drive as long as the impairment does not come to the authorities attention (Bro and Lindblom, 2018). In the Netherlands, the driver would be allowed a Practical Fitness to Drive Test. If drivers are able to show that they can compensate for the acuity loss in real traffic, they are allowed to hold their driver’s license. In all other countries, the driver of the mentioned case would stop driving after compulsory periodic vision screening when renewing the license.

The biggest discrepancy between countries can be seen in the list of investigations that are carried out at the basic medical examination for driving license. Slovenia was until recent quite strict in this regard, as it was one of the few countries that required tests of all visible functions to be performed. In February 2022, new law entered into force and since then, only visual acuity and visual field are requested to be checked by law for Group 1 drivers. In addition, since February 2022, refractive errors without other eye pathologies are no longer the reason for obligatory periodic testing as it used to be before, assuming that one is responsible for using accurate visual aids.

The limited duration of a medical certificate probably limits someone who does not have vision problems in everyday life, especially when the problems were related solely to refractive errors. On the other hand, many people who did not have any problems with a medical examination performed at the first testing, i.e., at the age of 18, do not need to perform other visual examinations until the age of 70, unless they decide to have an examination themselves. We know that visual function deteriorates with age and eye diseases, and so there are many people on the road who may no longer meet the criteria but still have a valid medical certificate, as they did not have to undergo medical examinations when their driving licenses were issued or renewed. At the same time, many people may have lost, or were issued limitations of, their driving licenses at the age of 70 due to very detailed and arduous testing of visual functions, even though they were driving the vehicle without any major problems in everyday life. These were the reasons that Slovenia just recently dropped the regulations that required comprehensive testing of additional visual functions at every renewal.

There are considerable ambiguities in investigations that are not compulsory required by a European directive and do not have clear exclusion criteria, especially as the testing equipment is not standardized. Many times, visual acuity or visual field results are poorer on occupational medicine testing systems. Especially elderly candidates frequently report more difficult and too fast testing on occupational medicine devices in comparison to standard tests in ophthalmology offices, by which these occupational medicine test results are usually verified. In addition to the expenses for the patients, such loops represent burden to the health system itself. There is no comparative data between the results on different occupational medicine devices and standard ophthalmological methods for testing of visual functions and that would be needed to make valid comparisons.

## Discussion

According to the above written justification of the importance of each visual function for safe participation in traffic, there is a need for better harmonization of the rules and procedures to assure safe driving across the whole EU territory and to come to equal treatment of all EU citizens. We aimed to present the current knowledge and regulations on one hand, and differences in their implementation in everyday practice in different European countries. A rather detailed comparison table has been prepared by the European Association of Opticians and Optometrists European Council of Optometry and Optics (2017), but there are probably some entries in this document that need to be updated. Thus, our comparison tables, to the best of our knowledge, represent the most current and up-to-date comparison of countries with each other and in deviation from the requirements of the EU directive, divided into individual visual functions.

Unlimited duration of driving licenses or just regular renewals without medical examination impose responsibility and self-criticism in assessing one’s own driving skills. The premise of unlimited medical certificates imply that an individual must order an ophthalmological examination in case of any vision problems, which the elderly often avoid because of fear for losing their driving licenses. Studies have found that the level of self-criticism toward one’s own abilities decreases with age and that as many as two thirds of drivers with severely impaired visual acuity overestimate their vision (Sandlin et al., 2014). On the other hand medical specialist have a duty to warn a patients about the condition and medical limitation on driving but is not allowed to share that information with other parties (like the police or the driving license authority) due to privacy-regulations. However, in some countries medical specialists are in the position to take away the license or even obliged to inform authorities if the driver does not fulfill the requirements. For that reason some drivers with eye diseases refuse to see an eye doctor and do not receive the medical treatment or medication they need. On the other hand, in the Netherlands, the law of privacy is more important than the law of driving, so doctor is not allowed to share medical information even if the driver does not fulfill a criterion. The Netherlands can also be an example of good practice, as they relate a lot on self-reporting of drivers. The education of drivers and use of self-reporting mostly resulted in awareness of patients who are well informed about their responsibilities, while the doctor’s role is to help them make the right choice and cease driving if necessary.

In many European countries, the mean age of the population is increasing, as more than 10% of the population is over 65 years of age. The absolute share of driving license holders has also seen a significant increase in the number of holders over the age of 65 over the last decade (Bilban, 2014).

On the other hand, there are probably many elderly people who undergo a vision examination in the seventies and eighties and may not pass it successfully due to overtly demanding examinations, even though they have no problems in everyday driving, or they have adapted their behavior so that the ride is still safe. In our ophthalmological practice, the opinion is often expressed that the tests performed on various occupational medicine devices are too demanding and that the restrictions imposed by them (e.g., permission to drive only during the day or short distances) are unfounded or too harsh. A common reason for the discrepancies may be the fact that the contrast sensitivity test must be performed with optimal correction, as otherwise the results are worse, and the driver’s ability may be inadequately assessed (Brovet Zupanèiè, 2014). For some elderly people, especially those who do not visit the ophthalmologist regularly, the necessary correction may not be precisely determined and may carry out such an investigation under inappropriate conditions. The European Association of Optometrists and Opticians European Council of Optometry and Optics (2017) is calling for a unification of the rules on who performs vision examinations. In some places they are performed in driving schools, in others they are performed by occupational medicine specialists or by ophthalmologists, whilst in some, they rely on the self-statements and self-criticism of candidates. It may thus happen that on the one hand on European roads, non-critical and dangerous drivers may drive with gradually progressing diseases (e.g., glaucoma or age-related macular degeneration), as they have no restrictions until the seventies or eighties. On the other hand, more self-critical or honest ones may be eliminated due to excessive requirements. The website of Portare Working Group (2009) can thus serve as a starting point for suggestions on how to perform certain tests and thus help to increase more unification.

The frequency or exclusion of periodic vision examinations and the obligation to assess the visual function upon renew of driving licenses therefore would need to be harmonized among EU countries so that all the countries would have the same regulations. Studies that compared the number of collisions in individuals with and without periodic examinations did not show significant differences in the number of collisions. An extensive European study by Van den Berg (2005) found that the prevalence of acuity problems and visual field loss in young people is low. Acuity problems rise to 5% in people over 75, but many meet the standards by wearing appropriate correction (Van den Berg, 2005). It is worth mentioning that EU-regulations allow drivers with the acuity worse than 0.5 but better than 0.16 to obtain licenses if they demonstrate proficiency in the use of bioptic telescope while driving (visual acuity must be at least 0.5 looking through a telescope) as well as meeting all other requirements. A bioptic telescope is an assistive device for individuals with low vision (Owsley, 2012). However, this is not effectuated in all EU-countries. On top of that, very few countries offer rehabilitation to train drivers on how to use optics for driving.

The prevalence of visual field problems is 2.7% in people over the age of 75, and it is even higher with contrast and glare sensitivity. It would be sensible to perform measurements of the last two functions, but the problem is that the rules do not yet define the norms for performing measurements and the criteria that an individual must meet. The opinion of the working group, which met in 2005 (van Rijn, 2005), as well as the European Association of Optometrists and Opticians in 2017, was that further research is needed to determine the limits, or acceptable values that the driver must have (“cut-off values”) in tests of contrast sensitivity, glare sensitivity and twilight vision. Since then, nothing has changed in the area of setting these values. However, when reviewing the literature in this field, the only one we noticed was that of the Irish College of Ophthalmologists (2021) which set a limit value according to the ICO standard of contrast sensitivity, adopted 1.5 for photopic and 1.2 for mesopic/glare for drivers in group 1.

There are also many questions and dilemmas regarding the unification of visual field measurements and the suitability of apparatus and methods for it. Esterman algorithm is a standard supra-threshold test that is accessible and comparable between Humphrey, Octopus, and some other perimeters. However, it does not fully comply with European directive visual field requirements. Additional problems are the differences in the definition of positive and negative results. In general, the duration of the perimetry testing should be balanced with the risk of losing the candidate’s concentration and focus. Because the number of test points is subject to limits, the visual field test contains a degree of uncertainty. Revocation of a driving license can have a significant negative impact on a person’s life and health, so a legal argument could be made that a positive test result should be unambiguous and reliable.

Another important step in harmonization of visual field requirements was made recently by Jørstad et al. (2021). The calculations that were considered in creating the new program compromise on sensitivity in favor of specificity, so that the risk of revoking a driver’s license on false premises is reduced to a minimum. Theoretically, a central test point distance of 6.5° and a lower limit of three adjacent missed test points provide a new perimetry algorithm with high specificity; that is, central scotomas up to the size of a physiological blind spot will not generate positive test results. A central test point distance of 6.5° results in 37 test points within 20, somewhat more than the 25 suggested by the Eyesight Working Group (van Rijn, 2005). Still, the sensitivity is limited, and only central scotomas with radii of at least 6.5° will always present positive test results. The sensitivity can be improved without compromising specificity by simultaneously reducing the central test point distance to 4.5° and increasing the limit of missed test points to four. However, this leads to a near doubling of central test points from 37 to 73, which increases the risk of fatigue and poor reliability. A greater test point distance outside 20° is likewise necessary to confine the total number of test points. An 8° peripheral test point distance limits the sensitivity for peripheral scotomas. Yet, it can be presumed to compromise the sensitivity for focal defects to a greater extent than more relevant peripheral findings, such as concentric or homonymous visual field loss (Jørstad et al., 2021).

It is good to keep in mind the fact that the same problem (measured value) does not always lead to the same functional problems, as individuals can compensate for them differently. Thus, in borderline cases, in addition to just measuring visual functions, it makes sense to perform a driving test, which shows how someone overcomes vision problems or whether it restricts candidate’s driving at all (van Rijn, 2005). After this test the driver’s ability to drive safely can be evaluated individually and a driving license with certain restrictions can be made. In Slovenia, drivers may exceptionally be allowed to drive when they do not meet the conditions for visual field or visual acuity referred to in the paragraphs before, if the ability to drive is determined by an occupational, traffic and sports medicine specialist at an authorized healthcare provider based on ophthalmologists specialist opinion and on a practical driving test. Such “allowances” have also recently been investigated in an international study (Sudmann et al., 2022) where considerable differences were found between different experts when evaluating borderline visual field defects.

The borderline drivers in EU countries are considered by special medical committees, but common guidelines are lacking. The purpose of individual assessment is to maintain at least partial safe mobility, which is most important for the quality-of-life relations. An individual assessment can, however, pose a problem of unequal treatment and subjectivity of the assessor, therefore some standards of harmonization is needed. In the case of borderline values, drivers may receive a restricted driving license. Such evaluations are complex and involve psychological and ophthalmological assessment. These are the reasons why borderline cases would best be tested in safe driving centers. The instructors that are informed about the leading problem would know what to pay special attention to. In Slovenia, such driving tests are carried out in several recognized safe driving centers. The candidates are mainly older drivers who are renewing their licenses. When testing, they drive with their own or rented vehicle. The test consists of driving in a closed part of the polygon and driving a vehicle in traffic. In the polygon part, they assess general driving abilities, such as starting, shifting and maintaining the direction of travel. This is followed by a braking exercise, where the candidate is evaluated according to the understanding of the instructions for the exercise, the number of repetitions of these instructions, braking points, braking intensity, achieving speed, and correcting driving direction when braking on different surfaces. This exercise is followed by a retreat exercise, where drivers withdraw first static (standing) and then dynamic (moving) obstacles. They examine if drivers perceive the difference between the two obstacles. This is followed by cornering, where drivers accelerate to the limit of vehicle slippage. They assess how drivers react in such a situation. If they are satisfied with the ride, the driver continues to drive in traffic, with the driver driving on local, regional and motorway roads. They mainly assess how many instructions drivers can remember, how they react to other participants, or monitor and follow the set traffic signals, parking (reverse, forward, and sideways). While driving, they assess how much the dialogue affects the maintenance of the direction and speed of driving. When driving on the motorway, they also test the driver’s vision with simplified approach. The driver is asked to read the registration number of overtaking vehicles in the tunnel in low light and in the open outer part of the motorway. The driver must be able to orient himself correctly and return to the safe driving center on his own. After the ride, candidates must evaluate their ride and list the mistakes they made while driving. For each test, they fill out a form, which is then forwarded to the referring doctor, who makes the final decision.

Similar practical driving tests are done in some other countries. However, the detailed comparison among all countries, included in this paper, was difficult as published legislations often do not mention how they are dealing with »borderline« cases, so we are not informed about official dealing with borderline cases in all of the countries. In Sweden, patients with visual field defects on testing can do a practical driving test on a driving simulator. In January 2018, 65% of drivers regained their license (Bro and Lindblom, 2018). In Norway, drivers can perform a Practical Fitness to Drive test on the road. Similarly, in the Netherlands, they only test in real traffic conditions, not on polygons. The final decision about fitness to drive is made by an expert on Practical Fitness to Drive of the Dutch driving license authority (CBR) and a medical doctor of the CBR. We believe similar tests are done in many other countries as well. Withaar et al. (2000) tried to validate different assessments of fitness to drive in older patients with cognitive impairment. After reviewing the literature, they concluded that on-road practical testing could be the best predictors of ability to drive safely. They suggested that on-road test is best administered in natural driving conditions with standard observation and scoring procedures. For such a scoring system, the relevant behaviors should be clearly described, enhancing interrater reliability. Attention should be paid to operational and tactical aspects and possibly to their interaction. Driving performance maybe rated on a differentiated scale with multiple items each ranging from, for example, 1 (insufficient) to 4 (good) (Withaar et al., 2000). They prepared Test Ride for Investigating Practical Fitness to Drive checklist (TRIP-score) that can be used when testing the candidate. There are many suggestions for protocols to be used when having a driver with specific visual impairment on PORTARE Working Group website which can be a helpful and welcoming step in harmonizing protocols around EU countries. For poorer visual acuity they suggest checking if the driver observes and reacts promptly and correctly in traffic. When testing the patients with nystagmus, the attention should be given on steadiness of steering, the ability to make timely observations, an automatic car may help them keeping more attention available for compensatory viewing technique. In cases of drivers with visual field deficits, the assessor should check if driver has any evidence of inattention, is aware of the visual field defect and reacts adequately, makes constant compensatory observations so that a timely response to (un)expected situations is possible. For checking the twilight vision, driver should do an on-road test during darkness or when light conditions are changing (from twilight zone to darkness) to check if the candidate can still behave sufficiently in these conditions. There are published protocols for monocularity and double vision as well.

While reviewing the literature, we also noticed a lack of studies made on polygons or simulators that would be necessary to set the limit values that actually affect the driving of the elderly. It is important to know that good and reliable test are necessary, and it remains a challenge to develop a relatively simple set of tests with a clear relation to driving safely and defined cut-off values. Having that in mind, it is important to harmonize the Practical Fitness to Drive among countries and educate employees of testing centers to be focused on the same problems and behaviors. Scialfa et al. (2011) developed a hazard perception test that present 18 short video scenes with an average testing time of 15 min and requires participants to indicate the presence of a traffic conflict that would lead to a collision between the »camera« vehicle and another road user. Results from their study suggested that this test of hazard perception could discriminate group that differ in driving experience (Scialfa et al., 2011). New and young drivers have longer responsive rates but then responsive time decrease with age until the mid-fifties. This is not surprising because hazard perception is visual and cognitive demanding skill that improves with experience. Benefits of training have been reported in studies on simulators and on road (Fisher et al., 2006; McKenna et al., 2006; Pradhan et al., 2009). It would be useful to explore further the hazard perception test as a screening tool for fitness to drive in older adults. The hazard perception test uses task-specific and realistic stimuli, so it is able to provide an objective assessment of drivers’ ability to receive and respond to genuine traffic conflicts (Horswill et al., 2008). If the driver performs poorly on the test, special training interventions aimed to improve their hazard perception skill could be offered (Horswill et al., 2008).

However, as much as it may be demanding, additional borderline evaluations should perhaps be standardized and harmonized for all EU countries as well.

## Conclusion

There is currently still rather large diversity in European countries in criteria to obtain or renew a driving license under the previous legislations and may not undergo regular examination until the late ages, unless they are monitored by an ophthalmologist for other eye diseases or because of subjective problems. This is where the importance of responsibility and self-criticism of each individual comes to the fore. Losing or restricting a driver’s license can be a severe psychological blow, as it has a major impact on the quality of daily life and access to health and other services and increases the elderly’s dependence on other carriers or public transport. Therefore, the plan should be to re-evaluate the current regulations and practices in the framework of cooperation between ophthalmology and occupational medicine, with common standards and exclusion criteria agreed upon. More practice with actual driving tests (on the road or in the simulator) to discriminate between safe and unsafe drivers with different types of visual field loss would need to be implemented in EU-countries with the use of Practical Fitness to Drive Tests. In addition, it is necessary to constantly raise awareness of vision and influence of aging on it among individuals and re-define the responsibilities of an ophthalmologist in the case of observed problems.

## Author contributions

NK and MH did the conceptualization of the article, collected and processed data, reviewed the manuscript, and agreed to publish it. NK prepared and wrote the manuscript. MH revised the manuscript and offered critical revisions and wrote the main part of discussion with proposals for the future. Both authors contributed to the article and approved the submitted version.
