# Supplementary material for: Comparison of visual requirements and regulations for obtaining a driving license in different European countries and some open questions on their adequacy
Source: Front Hum Neurosci. 2022 Sep 30;16:927712. doi: 10.3389/fnhum.2022.927712 (PMC9561926; doi:10.3389/fnhum.2022.927712)
Supplement: Supplementary file 1 [file Table_1.DOCX]

**Supplementary table 1.** References for data about requirements and rules regarding driving licenses in European countries and UK.

| EU Directive | EUR-lex. (2018). Direktiva 2006/126/ES Evropskega parlamenta in sveta o vozniških dovoljenjih. <https://eur-lex.europa.eu/legal-content/SL/TXT/PDF/?uri=CELEX:02006L0126-20180722&from=EN> [Accessed Nov 17, 2020]. |
| --- | --- |
| Slovenia | Pis. (2022). Pravilnik o zdravstvenih pogojih voznikov motornih vozil. [http://www.pisrs.si/Pis.web/pregledPredpisa?id=PRAV13322#](http://www.pisrs.si/Pis.web/pregledPredpisa?id=PRAV13322) [Accessed Apr 2, 2022]. |
| Italy | Automobile Club d'Italia. (2021). Regolamento Art.119. <https://www.aci.it/i-servizi/normative/codice-della-strada/titolo-iv-guida-dei-veicoli-e-conduzione-degli-animali/art-119-requisiti-fisici-e-psichici-per-il-conseguimento-della-patente-di-guida/regolamento-art-119.html?fbclid=IwAR1HLieLt9qDOuwkaeOhLthjND3oUJICqrIkhScHqKC3LqiyO7Spp0wt40E> [Accessed Oct 14, 2021]. |
| Croatia | Cadial. (2021). Zakon o sigurnosti prometa na cestama (NN 067/2008). <https://sredisnjikatalogrh.gov.hr/cadial/searchdoc.php?action=search&lang=hr&query=%22Zakon+o+sigurnosti+prometa+na+cestama+%28NN+067%2F2008%29%22&searchText=on&searchTitle=on&resultdetails=basic&filteracttype=all&filterfields=all&filtereuchapter=all&resultlimitnum=10&bid=RFjb%2bCBapBjvRsxALchsmA%3d%3d&annotate=on>  [Accessed Oct 14, 2021]. |
| Hungary | Wolters Kluwer. (2021). 13/1992. (VI. 26.) NM rendelet a közúti járművezetők egészségi alkalmasságának megállapításáról. <https://net.jogtar.hu/jogszabaly?docid=99200013.nm> [Accessed Oct 17, 2021]. |
| Austria | RECHTSINFORMATIONSSYSTEM DES BUNDES. (2022). Bundesrecht konsolidiert: Gesamte Rechtsvorschrift für Führerscheingesetz-Gesundheitsverordnung, Fassung vom 06.04.2022. <https://www.ris.bka.gv.at/GeltendeFassung.wxe?Abfrage=Bundesnormen&Gesetzesnummer=10012726> [Accessed Apr 3, 2022]. |
| Germany | Bundesministerium der Justiz. (2021). Verordnung über die Zulassung von Personen zum Straßenverkehr (Fahrerlaubnis-Verordnung - FeV)  Anlage 6 (zu den §§ 12, 48 Absatz 4 und 5)  Anforderungen an das Sehvermögen. <https://www.gesetze-im-internet.de/fev_2010/anlage_6.html> [Accessed Oct 17, 2021]. |
| The Netherlands | Overheid.nl. (2022). Regeling eisen geschiktheid 2000. <https://wetten.overheid.nl/BWBR0011362/2021-07-01> [Accessed Apr 3, 2022]. |
| Denmark | Sundhed.dk.(2022). 3 Synet (A) og hørelsen (B). <https://www.sundhed.dk/content/cms/92/102992_synskrav-p-16-22.pdf> [Accessed Apr 4, 2022]. |
| Switzerland | Schweizerische Eidgenossenschaft. (2021). Verordnung  über die Zulassung von Personen und Fahrzeugen  zum Strassenverkehr. <https://www.fedlex.admin.ch/eli/cc/1976/2423_2423_2423/de> [Accessed Oct 17, 2021]. |
| France | Republique Francaise. (2022). Journal officiel électronique authentifié n° 0079 du 03/04/2022. <https://www.legifrance.gouv.fr/download/pdf?id=8dD3wEzkeHMp59Q_y7Jrp2jXbwEqgi4p1G3fTjlpsFU=> [Accessed Apr 4, 2022]. |
| United Kingdom | Gov.UK. (2021). Driving eyesight rules. <https://www.gov.uk/driving-eyesight-rules?fbclid=IwAR0vXLNnVBr6rqsfNizHDU_DeEcxfxnWBUvjEIeWaxRvU87WBhcrTyxXPRQ> [Accessed Oct 17, 2021]. |
| Sweden, Norway, Iceland | Bro, T., and Lindblom, B. (2018). Strain out a gnat and swallow a camel? - vision and driving in the Nordic countries. Acta Ophthalmol. 96(6), 623–630. doi: 10.1111/aos.13741 |
